# Attitudes of Teenage Mothers towards Pregnancy and Childbirth

**DOI:** 10.3390/ijerph17041411

**Published:** 2020-02-21

**Authors:** Agnieszka Bałanda-Bałdyga, Anna Bogusława Pilewska-Kozak, Celina Łepecka-Klusek, Grażyna Stadnicka, Beata Dobrowolska

**Affiliations:** 1 Department of Development in Obstetrics, Faculty of Health Sciences, Medical University of Lublin, 20-081 Lublin, Poland; agnieszkabalanda@wp.pl (A.B.-B.); grazyna.stadnicka@umlub.pl (G.S.); 2 Chair and Department of Gynaecology and Gynaecological Endocrinology, Faculty of Health Sciences, Medical University of Lublin, 20-049 Lublin, Poland; anna.pilewska-kozak@umlub.pl (A.B.P.-K.); celina.lepecka-klusek@umlub.pl (C.Ł.-K.); 3 Department of Development in Nursing, Faculty of Health Sciences, Medical University of Lublin, 20-081 Lublin, Poland

**Keywords:** attitude, pregnancy, childbirth, teenage mothers, dispositional optimism

## Abstract

The problem of early motherhood is still a serious medical and social problem in many countries around the world. The aim of this study was to analyze the attitudes of teenage mothers towards pregnancy and childbirth. A retrospective cross-sectional study was conducted with the use of an original questionnaire containing a test to measure attitudes on a five-point Likert scale and a Life Orientation Test-Revised (LOT-R) to assess dispositional optimism. The study involved 308 teenage mothers between 13 and 19 years of age. Attitudes of teenage mothers towards pregnancy and childbirth were more often positive (90.6%) than negative (9.4%). Sociodemographic features determining the attitudes of teenage mothers towards both their pregnancy and childbirth included their age, marital status, current occupation, and main source of income. The type of attitude adopted by teenage mothers towards pregnancy and childbirth was significantly related to the level of their dispositional optimism.

## 1. Introduction

The occurrence of pregnancy in a teenager disturbs the commonly accepted model of life, in which the first step is to obtain an education, take up a job and become financially independent, and only then start a family [1,2,3]. It has an adverse effect on the personal sphere of life (health and emotional stability), as well as on the social aspect of life. Teenagers are standing on the threshold of adult life with no life stabilization in terms of job security, own habitation, and financial and economic independence. They face difficulties in continuing education, are dependent on their parents who exercise custody, and have a harder start on the labor market. It is less likely for a pregnant teenager or a teenage mother to get employed; they are offered fewer working hours and lower salaries in comparison to women who gave birth to their first child in adulthood [4,5,6,7,8]. From a medical point of view, the young age of a mother increases the risk of abnormalities in the course of pregnancy and childbirth and in the condition of the newborn [9]. It is worth noting that, despite extensive research on the subject, opinions of researchers are still divided. Authors link negative obstetric outcomes to poor living conditions of girls [6,10,11,12], risky health behaviors [10,13,14], inadequate prenatal care [9,14,15,16,17], and biological immaturity [15,18]. There is no controversy, however, over the observation that the younger the mother, the greater the risk of complications in her and her child [9,19].

Pregnancy and childbirth are extremely important events. They initiate a new stage in the life of a woman and her family. Most women of reproductive age prepare for pregnancy and childbirth both physically and mentally. From medical, psychological, sociological, and legal point of view, the arrival of a new life, together with the whole period of pregnancy, childbirth, and puerperium, seems to be one of the most important events, which has an impact on the whole future of a woman, her child, and her family [20,21]. Various factors of personal and social life have a significant influence on the final shape of the attitudes of contemporary women towards pregnancy and childbirth, especially among teenage mothers [1,8,14,22,23].

As a result of inadequate sex education, difficult life situation, and a tempestuous period of adolescence, many girls below the age commonly consider it appropriate to engage in sexual activity with often equally immature partners, which frequently results in an unplanned pregnancy. In professional literature, it is sometimes referred to as ‘time of crisis’ or off-time mothering, which occurs too early according to the biological, social, and psychological rhythm. It causes changes in girls’ personalities and influences their system of values and their plans for the future [1,24]. 

The problem of teenage motherhood has been raised in some studies conducted by foreign investigators. They did not, however, concentrate on the already existing pregnancy and childbirth, but focused on the attitudes of teenagers towards a possible pregnancy [25]. Other studies investigated, for example, the impact of teenage motherhood on the socio-economic situation in their adulthood [7], on interpersonal relations of teenage mothers [26], and on their educational achievements and earnings [27]. There were also studies that focused on the functioning of girls who had their first child in their teens and are still in their teenage years. In these studies, investigators analyzed the needs of pregnant teenagers and teenage mothers, and the attitude of these girls to the child during pregnancy and after giving birth [28]. Researchers also studied reactions of teens and their parents to the pregnancy news and the situation of teenage parents after the birth of a child [29], social situation of teenage mothers [30], preparation of teenage girls to single parenthood [31], and correlation between experiencing first motherhood in teenage years and more negative parental attitudes and a lower level of self-esteem [1]. The results of the aforementioned studies and the lack of available empirical research on the attitudes of teenage mothers towards their own experience of maternal events triggered the current study, which aimed to examine the attitudes of teenage mothers towards pregnancy and childbirth and to outline most important determinants of these attitudes. The authors adopted the definition of attitude by Zimbardo and Leippe, according to which it is a learned, relatively constant tendency to a positive or negative assessment of a person, concept, or event [32].

## 2. Materials and Methods

A cross-sectional study was carried out among a convenience sample of 308 teenage mothers from 8 purposefully chosen hospitals from central and eastern Poland. The study group comprised teenage mothers between the first and the third day postpartum. The inclusion criteria were: age up to 19 years, good overall health condition at the time of the survey (i.e., normal limits of the general parameters, such as body temperature, pulse rate, and blood pressure, and normal limits of the obstetrical parameters, such as lactation, the height of the pelvic floor, and lochia), willingness to participate in the study, written informed consent for participation in the study, and, in the case of minors, also the consent of their legal guardians. 

The study was retrospective, and participation was voluntary and anonymous. Out of 328 girls invited to participate in the study, 5 did not grant the consent without giving a reason, and 15 submitted incomplete questionnaires, so these 20 girls were discarded in this study. A request for assistance in carrying out the survey was addressed to certified professional midwives holding the position of ward sisters in the Department of Obstetrics. Before starting the survey, the midwives were given instructions on how to fill in the questionnaire, and, in the case of a minor, also her legal guardian was instructed. Respondents could ask for additional explanations, and there was no time limit imposed. They were also reassured that they could resign from the participation in the study at any time. Respondents were asked to leave the completed questionnaires in an unsigned envelope in the midwives’ room.

The research tool was an original questionnaire designed for the purpose of this study. It included a test to measure the attitudes of women towards pregnancy and childbirth. It also contained questions related to the age of respondents, their overall social and living conditions, as well as a Life Orientation Test.

The test to measure attitudes was adopted from the literature [33,34]. It contained 16 statements, half of which concerned the pregnancy and the other half the childbirth (Table 1).

The researchers used the typical Likert scale with five possible answers: I strongly agree, I agree, I neither agree nor disagree, I disagree, I strongly disagree. Positive statements (1,3,5,7,9,11,13,15) were assessed in the following way: I strongly agree—5 points, I agree—4 points, I neither agree nor disagree—3 points, I disagree—2 points, I strongly disagree—1 point. The reverse scores were applied to negative claims (2,4,6,8,10,12,14,16). The maximum number of points in each part (pregnancy and childbirth) was 40. A score of up to 16 points meant a negative attitude, and above 16 points meant a positive attitude. The Cronbach’s alpha reliability coefficient was 0.76 for the pregnancy subscale and 0.82 for the childbirth subscale. The mean correlation between the statements was 0.3 and 0.37, respectively. 

Life Orientation Test-Revised (LOT-R) developed by Scheier, Carter, and Bridges, in the Polish adaptation of Poprawa and Juczyński [35], is used to measure dispositional optimism expressing generalized expectations concerning positive events. It is intended for healthy and unhealthy people. It consists of 10 statements, six of which have a diagnostic value for dispositional optimism. The overall test result is the sum of the evaluation of six statements, including three positive and three negative. Possible results are in the range of 0–24 points. The higher the score, the higher the level of optimism. The reliability determined by the Cronbach’s alpha coefficient is 0.78 for the original version and 0.76 for the Polish version. The raw scores are converted to standard sten scores to enable the assessment of the intensity of the dispositional optimism. A score of 0–12 points (1–4 sten) identifies people who tend to be pessimistic; 13–16 points (5–6 sten) indicate moderate optimism; 17–24 points (7–10 sten) indicate optimism [35].

The values of the analyzed parameters measured on a nominal scale were characterized by the number and percentage, and the ones measured on a ratio scale were characterized by the mean value and lower and upper quartile with the range of variability. Homogeneity or independence χ² tests were used to assess the existence of differences or dependencies between the analyzed parameters. For small data sample in the studied subgroups (below 5), the Yates correction was used. A 5% inference error and the associated materiality level *p* < 0.05, indicating the existence of statistically significant differences or dependencies, were assumed. Statistical analyses were performed using STATISTICA software version 10.0 (StatSoft, Lublin, Poland).

The research project was positively assessed by the Bioethics Committee of the Medical University of Lublin (Resolution No. EC—0254/157/2012 of 28 June 2012).

## 3. Results

### 3.1. Characteristics of Respondents

The age of the respondents ranged from 13 to 19 years, with an average of 18.1 (Me 19; Q_1_ 17; Q_3_ 19). The youngest age group (up to 15 years of age) counted 7 (2.3%) girls, the medium age group (16–17 years of age) comprised 79 (25.6%) girls, and, in the oldest group (18–19 years of age), there were 222 (72.1%) girls. A total of 152 (49.4%) respondents were in a common-law partnership with the father of the child, 90 (29.2%) were married, and 66 (21.4%) were single mothers. Over half of the respondents were rural residents (176; 57.1%), and the remaining 132 (42.9%) teenagers lived in cities. In this group, 82 (26.7%) lived in cities with a population of under 200,000 inhabitants, and 50 (16.2%) lived in cities with a population of over 200,000 inhabitants. Most of them went to schools (207; 67.2%), 23 (7.5%) worked, and 78 (25.3%) did neither. At the time of the survey, most respondents were financially dependent on their parents (137; 44.5%) or on the father of the child and his family (136; 44.1%). Nineteen (6.2%) were self-sufficient, and, for the remaining 16 (5.2%) girls, the source of income was unemployment benefit, child maintenance, or a social assistance benefit from the municipal social welfare center. For 275 (89.3%) respondents, it was their first pregnancy, for 28 (9.1%), that was the second pregnancy, and the remaining 5 (1.6%) girls had been pregnant two or more times before. A total of 211 (68.5%) respondents claimed that the pregnancy was not planned. The other 97 (31.5%) declared that they had planned the pregnancy. All the pregnancies were single. A total of 229 (74.4%) teenagers had a natural birth, the remaining 79 (25.6%) had a cesarean section. The most frequent indications for the cesarean section were: failure to progress, fetal rhythm abnormalities, and difficult cooperation with the parturient. 

### 3.2. Attitudes Towards Pregnancy and Childbirth

The test used in the study allowed to identify girls with a positive and negative attitude towards pregnancy and childbirth. The minimum number of points scored in the test for measuring women’s attitudes towards pregnancy was 12, and the maximum score was 37 (Me 25.0; Q_1_ 22.0; Q^3^ 28.5), and towards childbirth 8 and 40, respectively (Me 26.0; Q_1_ 23.0; Q_3_ 29.5). The relationship between the presented types of attitudes is shown in Table 2.

A positive attitude towards pregnancy and childbirth was presented by 87.0% of the respondents and a negative attitude to these both experiences by 5.8%. A positive attitude to pregnancy and a negative attitude to childbirth was characteristic for 3.6% of the respondents, and also 3.6% of girls presented a negative attitude to pregnancy and a positive attitude to childbirth. The relationship between the presented types of attitudes towards pregnancy and towards childbirth was statistically significant (*p* < 0.00001). The relationship between attitudes towards pregnancy and childbirth and the respondents’ age, marital status, place of living, current occupation, and the main source of income is shown in Table 3. 

The type of attitude towards pregnancy was significantly related to the age (*p* = 0.00001), marital status (*p* = 0.0003), current occupation (*p* = 0.007), and the main source of income (*p* = 0.00001) of the respondents. Their place of living proved insignificant (*p* = 0.29). A similar significant relationship was also present in the attitudes of girls towards childbirth and their age (*p* = 0.0006), marital status (*p* = 0.003), and the main source of income (*p* = 0.0002). The current occupation was borderline statistically significant (*p* = 0.05). The place of living, on the other hand, was not statistically significant in this respect (*p* = 0.75). 

The minimum number of points scored in Life Orientation Test-Revised (LOT-R) was 3, and the maximum was 23 (Me 14.0; Q_1_ 12.0; Q_3_ 17.0). Moderate optimism (5–6 sten) was observed in 126 (40.9%) girls, 83 (26.9%) girls were optimistic (7–10 sten), and a tendency to being pessimistic (1–4 sten) was found in 99 (32.2%) women. Table 4 presents a summary of data on the attitudes of the respondents towards pregnancy and childbirth, depending on the level of optimism. 

The analysis showed a significant correlation between the level of optimism and the attitude towards pregnancy and also the attitude towards childbirth: *p* = 0.02; *p* = 0.005, respectively. Girls showing a tendency to be pessimistic presented negative attitudes towards pregnancy and childbirth more frequently.

## 4. Discussion

Based on reports from the literature, it could be assumed that many teenagers adopt a negative attitude towards pregnancy and childbirth [1,3,28]. The test to measure attitudes used in this study did not confirm this. Negative attitudes towards maternal events were detected only in 9.4% of girls. The vast majority (90.6%) had a positive attitude towards pregnancy, as well as childbirth, and the relationship between the presented attitudes was significant (*p* < 0.00001). Moreover, most of the variables adopted in this study, except for the place of living, significantly differentiated the numerical distribution of the surveyed characteristics (*p* < 0.05). 

It is interesting that all teenagers whose pregnancy ended with a cesarean section (25.6%) had positive attitudes towards childbirth. The problem arose as to which birth method this result should be referred to—the vaginal delivery or the childbirth by surgical means. Clarification of this issue requires separate research as it is not known how many respondents had an elective cesarean section ‘on request’. The hospital documentation of the respondents shows that the performed c-sections had always had medical indications, and only sometimes also psychological (e.g., difficulties in establishing cooperation with the parturient). According to reports in the literature, vaginal delivery is a stressful experience for women, mainly because of pain. Therefore, they prefer to deliver the baby by cesarean section, even without medical indications [36,37,38,39,40,41]. Moreover, some young women, who have not yet experienced childbirth, build their ideas about the natural labor on the basis of information taken from unprofessional sources—from their mothers, grandmothers, and friends. Consequently, they fear not only pain but also many hours of exhaustion and possible complications [28]. They also develop a conviction that surgical childbirth is safer (for them and for the baby) than natural labor [36,37,42]. These factors might have influenced the attitudes of teenage mothers who took part in the study and resulted in positive attitudes towards the cesarean section.

The Polish authors, whose research tool was used in our study, studied the attitudes of women towards pregnancy and childbirth with an aim at showing differences in prevalence of negative and positive attitudes, depending on the method of conceiving a child—natural or assisted, and they showed that the attitudes towards pregnancy were significantly more often positive than negative (*p* < 0.05), and, towards childbirth, the tendency was reversed [33,34,43]. However, the results of their study could not be compared with the results obtained in this current analysis because it was conducted among adult women, married, living in fairly good socio-economic conditions, and their pregnancies were planned, desired, and expected. Moreover, it must be noted that negative attitudes expressed by some respondents who participated in the mentioned study could be explained by the fact that achieving success in infertility treatment does not oblige a woman to treat her pregnancy as a joyful experience. The results of another study, conducted by Hall et al., suggested that attitudes towards motherhood should be considered in a broad context, including the physical and mental health of women and the social environment in which they live [12].

Authors who conducted research among sexually active girls examined attitudes not in relation to the already existing pregnancy and experienced childbirth, as it was done in this study, but in relation to a potential pregnancy [25,44]. Anyway, the analysis of the collected material showed that 20% of girls had a definitely negative attitude towards a possible pregnancy, 8% had a definitely positive attitude, 14% were indifferent, and, in other cases (52%), it could not be determined. Perhaps difficulties, which over half of the respondents had in determining their attitude, resulted from the fact that at the time of the research, they had not experienced pregnancy yet and simply could not relate that situation to themselves. 

Motherhood is an important and momentous event in the life of every woman. However, it has a different meaning to a woman who becomes a mother at the age of early school education than to a woman who becomes a mother at a later stage of life [45]. The division into age groups adopted in this study significantly differentiated the attitudes of the respondents towards both pregnancy (*p* = 0.00001) and childbirth (*p* = 0.0006). It is surprising that all girls from the youngest age group (13–15 years old) presented positive attitudes towards these events. It is not possible to explain the reasons for such a positive perception of this new, objectively difficult situation by the youngest mothers on the basis of the collected material. It would require separate research. Some authors concluded from the material they analyzed that very young pregnant women had a positive perception of their pregnancy, expecting that the child would have a positive impact on their relations with the child’s father and that their pregnancy would strengthen family ties and improve social conditions [6,44,46]. If and how real it is, we don’t know. It is only known that such a situation quite often increases the probability of raising a child without the involvement of the father [47]. 

Optimism, as one of the personal resources, promotes effective coping with stress, which makes it pro-healthy because stress is one of the factors that negatively influence psychophysical well-being [48,49]. A high level of dispositional optimism makes it easier for people to adapt to new living conditions, prevent helplessness, increase the sense of happiness [49]. This has been confirmed in the presented study because girls who had a tendency to be pessimistic presented negative attitudes towards pregnancy and childbirth more frequently.

The study had several limitations, preventing the generalization of conclusions. Firstly, there was no control group. The establishment of a control group in this kind of study poses a number of problems. These problems were taken into account when planning the study, and, eventually, the investigators decided not to collect study material from a control group. First and foremost, a control group comprising adult mature mothers would make it impossible to compare the tested variables in relation to teenage motherhood. On the other hand, taking potential teenage mothers as the control group, which means conducting a survey among teenagers who are not pregnant and who have not experienced childbirth (such as in the study by Brucner and co-authors [25]), would not refer to the reality of pregnancy and childbirth as own experience, but only to a purely hypothetical situation. 

It would be a good idea to form a control group out of teenage mothers who gave birth a few weeks earlier, as their hormonal levels would be stabilizing. However, it would be very difficult to collect the study material from them because teenage mothers often do not have a permanent residence, which makes it hard to contact and reach such respondents. 

Another limitation of this study is the fact that the material was collected 1–3 days after giving birth, at the time of huge hormonal changes, which might affect the attitudes of women towards motherhood. In this study, negative attitudes towards maternal events were detected in 9.4% of girls, and, in the case of this group, the question arises whether the result was not influenced, among other factors, by their hormonal imbalance.

The presented study material proved that the attitudes of teenage mothers towards such important maternal events as pregnancy and childbirth are a vast and complex problem. In order to find out more about it and understand it better, further research, especially of an interdisciplinary and qualitative nature, is needed. An in-depth quantitative and qualitative analysis, carried out by researchers representing different scientific disciplines, can contribute to the optimization of counseling offered to sexually active young people, with respect to diversities in world views and standpoints. It is also to be hoped that a better understanding of this issue will facilitate communication between medical personnel and teenage patients and between teenagers, parents, and teachers and that it will enable the development of support strategies in the situation of teenage motherhood, as pointed out by other researchers [8,21,24].

## 5. Conclusions

1. Attitudes of teenage mothers towards pregnancy and childbirth varied but were much more frequently positive than negative.
2. Sociodemographic characteristics, which determined the attitudes of teenage mothers towards both pregnancy and childbirth, included their age, marital status, current occupation, and main source of income.
3. The type of attitude adopted by teenage mothers towards pregnancy and childbirth was significantly related to the level of their dispositional optimism.

## Figures and Tables

**Table 1:** Statements evaluated on a five-point Likert scale.

Time	Statements
Pregnancy	Pregnancy is a time for a woman to happily await the birth of a child. Pregnancy is a time of fear and anxiety for a woman. Pregnancy is a time of good relations in a partnership and in the family. Pregnancy is a time of disorganization of a woman’s personal life. During pregnancy, a woman experiences many interesting maternal experiences. Pregnancy requires a great deal of sacrifice from a woman. Pregnancy is a time when a woman feels more valued than ever before. Pregnancy is a difficult time for a woman—full of sacrifices.
Childbirth	9. Childbirth experience boosts a woman’s self-esteem. 10. Childbirth is a difficult time that a woman wants to forget quickly. 11. Childbirth is a time of intense maternal experience. 12. The childbirth experience of women is dominated by pain. 13. Childbirth is a partnership and family celebration. 14. Childbirth is a nightmare for a woman. 15. Childbirth is a joyful and moving event for a woman. 16. Childbirth is devoid of maternal experience.

**Table 2:** The relationship between attitudes towards pregnancy and childbirth.

Attitude		Towards Childbirth			
		Negative n = 29; 9.4%		Positive n = 279; 90.6%	
		N	%	n	%
**Towards pregnancy**	**Negative n = 29; 9.4%**	18	5.8	11	3.6
	**Positive n = 279; 90.6%**	11	3.6	268	87.0
**Statistical significance**		χ^2^ = 104.06; *p* < 0.00001			

**Table 3:** Attitudes towards pregnancy and childbirth and sociodemographic variables.

Variables		Attitude							
		Towards Pregnancy				Towards Childbirth			
		Negative n = 29; 9.4%		Positive n = 279; 90.6%		Negative n = 29;9.4%		Positive n = 279;90.6%	
		n	%	n	%	n	%	n	%
Age	13–16	0	0	7	2.5	0	0	7	2.5
	16–17	18	62.1	61	21.9	16	55.2	63	22.6
	18–19	11	37.9	211	75.6	13	44.8	209	74.9
Statistical significance		χ^2^ = 22.46; *p* = 0.00001				χ^2^ = 14.90; *p* = 0.0006			
Marital status	Single	15	51.8	51	18.3	12	41.4	54	19.4
	In common-law partnership	13	44.8	139	49.8	15	51.7	137	49.1
	Married	1	3.4	89	31.9	2	6.9	88	31.5
Statistical significance		χ^2^ = 22.46; *p* = 0.00001				χ^2^ = 11.44; *p* = 0.003			
Place of residence	Big city	2	6.9	48	17.2	5	17.2	45	16.1
	Small city	10	34.5	72	25.8	6	20.7	76	27.2
	Village	17	58.6	159	57.0	18	62.1	158	56.7
Statistical significance		χ^2^ = 2.47; *p* = 0.29				χ^2^ = 0.57; *p* = 0.75			
Current occupation	School	27	93.1	180	64.5	25	86.2	182	65.2
	Work	0	0	23	8.2	0	0	23	8.3
	Neither school nor work	2	6.9	76	27.2	4	13.8	74	26.5
Statistical significance		χ^2^ = 9.88; *p* = 0.007				χ^2^ = 5.79; *p* = 0.05			
Main source of income	Parents	26	89.7	111	39.8	24	82.8	113	40.5
	Father of the child	3	10.3	133	47.7	5	17.2	131	47.0
	Work	0	0	19	6.8	0	0	19	6.8
	Unemployment/social benefit	0	0	16	5.7	0	0	16	5.7
Statistical significance		χ^2^ = 26.61; *p* = 0.00001				χ^2^ = 19.43; *p* = 0.0002			

**Table 4:** The level of optimism and attitudes towards pregnancy and childbirth.

The Level of Optimism	Attitude							
	Towards Pregnancy				Towards Childbirth			
	Negative n = 29; 9.4%		Positive n = 279; 90.6%		Negative n = 29; 9.4%		Positive n = 279; 90.6%	
	n	%	n	%	n	%	n	%
Tendency to being pessimistic (1–4 sten) n = 99; 32.2%	16	16.2	83	83.8	17	17.2	82	82.8
Moderate optimism (5–6 sten) n = 126; 40.9%	7	5.6	119	94.4	7	5.6	119	94.4
Optimistic attitude (7–10 sten) n = 83; 26.9%	6	7.2	77	92.8	5	6.0	78	94.0
Statistical significance	χ^2^ = 7.95; *p* = 0.02				χ^2^ = 10.20; *p* = 0.005			
